# Roles of ROS and NO in Plant Responses to Individual and Combined Salt Stress and Waterlogging

**DOI:** 10.3390/antiox14121455

**Published:** 2025-12-03

**Authors:** Taufika Islam Anee, Nasser A. Sewelam, Nonnatus S. Bautista, Takashi Hirayama, Nobuhiro Suzuki

**Affiliations:** 1 Department of Materials and Life Sciences, Faculty of Science and Technology, Sophia University, Chiyoda, Tokyo 102-8554, Japan; 2 Department of Agronomy, Faculty of Agriculture, Sher-e-Bangla Agricultural University, Dhaka 1207, Bangladesh; 3 Botany Department, Faculty of Science, Tanta University, Tanta 31527, Egypt; sewelam@science.tanta.edu.eg; 4 Institute of Biological Sciences, College of Arts and Sciences, University of the Philippines Los Baños, Laguna 4031, Philippines; nsbautista1@up.edu.ph; 5 Institute of Plant Science and Resources, Okayama University, 2-20-1 Chuo, Kurashiki 710-0046, Okayama, Japan

**Keywords:** chloroplasts, mitochondria, nitric oxide (NO), reactive oxygen species (ROS), salt stress, stress combination waterlogging

## Abstract

During the climate change era, plants are increasingly exposed to multiple environmental challenges occurring simultaneously or sequentially. Among these, salt stress and waterlogging are two major factors that severely constrain crop productivity worldwide and often occur together. To survive under such conditions, plants have evolved sophisticated systems to scavenge harmful levels of reactive oxygen species (ROS). Despite their cytotoxic potential, ROS also act as key signaling molecules that interact with nitric oxide (NO), Ca^2+^, protein kinases, ion homeostasis pathways, and plant hormones. These signaling and acclimatory mechanisms are closely associated with the functions of energy-regulating organelles—chloroplasts and mitochondria—which are major sources of ROS under both individual and combined stresses. While many of these responses are shared between salt stress, waterlogging and their combination, it is likely that specific signaling mechanisms are uniquely activated when both stresses occur together—mechanisms that cannot be inferred from responses to each stress alone. Such specificity may depend on precise coordination among organelle-derived signals and the tight regulation of their cross-communication. Within this network, ROS and NO likely serve as central hubs, fine-tuning the integration of multiple signaling pathways that enable plants to adapt to complex and fluctuating stress environments.

## 1. Introduction

Salt stress is a major environmental challenge threatening global agriculture, affecting approximately 20% of the world’s irrigated lands [1]. Its impact is intensified by environmental degradation, poor irrigation practices, and climate change [2,3]. Enhancing plant tolerance to salt stress is thus a central focus in plant biology and crop science [4]. Salt stress imposes both ion toxicity and osmotic stress, leading to impaired key physiological processes and growth inhibition [5,6,7,8]. Excessive Na^+^ interferes with ion uptake and metabolism, particularly affecting K^+^-dependent enzymes that are highly sensitive to elevated Na^+^/K^+^ ratios [9]. Salt stress also disrupts energy metabolism involving chloroplasts and mitochondria. Elevated Na^+^ restricts water absorption, leading to osmotic stress and stomatal closure [9], which decreases CO_2_ availability through stomatal and mesophyll limitations [9]. Under limited CO_2_ fixation, excess reducing power accumulates, promoting oxidative damage in photosystems. The reaction center proteins in photosystem II are considered to be especially sensitive to such an impact caused by salt stress [10]. Salt stress also disrupts mitochondrial electron transport [11], which may be linked to morphological changes such as increased mitochondrial size [12]. These effects on energy-producing organelles are closely linked to overproduction of reactive oxygen species (ROS), which, at high levels, damage proteins, lipids, carbohydrates, and DNA, leading to cellular and organellar injury [13,14,15,16]. Indeed, increased ROS accumulation, lipid peroxidation (evaluated by malondialdehyde; MDA), membrane leakage, and growth inhibition under salt stress have been reported in crops such as rice, maize, tomato, and sweet pepper [17].

Waterlogging is another major environmental challenge that reduces crop yields, driven largely by poor drainage and land-use changes [18]. It occurs when soil pores become saturated, restricting oxygen diffusion and creating hypoxic or anoxic conditions [19]. Waterlogging consists of two phases: hypoxia (waterlogged) and reoxygenation (post-waterlogging) [20]. During hypoxia, limited oxygen shifts metabolism from aerobic to less-efficient anaerobic respiration, leading to energy depletion, nutrient imbalance, and accumulation of excess ROS [21]. Mitochondria are particularly sensitive, as impaired electron flow in complexes I and III causes electron leakage and ROS overproduction [22]. In addition, waterlogging restricts CO_2_ as well as O_2_ diffusion in roots and stems, resulting in photosynthetic inhibition and ROS generation in chloroplasts [23]. The reoxygenation phase can be even more damaging, as the sudden exposure to oxygen and light triggers oxidative bursts, exacerbating cellular damage and cell death [24].

To counteract ROS toxicity, plants employ integrated antioxidant systems comprising both non-enzymatic components; such as ascorbic acid (AA), glutathione (GSH), α-tocopherol, carotenoids, flavonoids, phenolics, and proline, and enzymatic antioxidants; including superoxide dismutase (SOD), peroxidase (POD), catalase (CAT), glutathione reductase (GR), ascorbate peroxidase (APX), monodehydroascorbate reductase (MDHAR), and dehydroascorbate reductase (DHAR) [15]. Within the networks, the ascorbate–glutathione (ASA–GSH) cycle plays a central role in maintaining redox homeostasis and efficiently scavenging ROS in coordination with enzymatic antioxidants [25,26]. These enzymes function in an interconnected manner, enabling compensation when one activity is reduced. Regulation of antioxidant systems has been linked to stress tolerance. For example, in maize, the suppression of miR169q under salt stress enhanced expression of its target gene encoding NUCLEAR FACTOR Y A8 (NF-YA8) and the antioxidant gene encoding PEROXIDASE 1 [27]. In rice, kinase- and phosphatase-mediated regulation of CAT activity contributed to the enhancement of salt tolerance [28,29]. Non-enzymatic antioxidants, such as AA, GSH, anthocyanin, and tocopherol, were also shown to mitigate salt-induced damage [30]. Upregulation of these enzymatic and non-enzymatic antioxidants and their contributions to protection of cells against oxidative damage have also been observed under waterlogging and related stresses, including flooding [31,32]. The importance of ROS-scavenging capacity in waterlogging tolerance is further supported by comparisons between sensitive and tolerant sesame lines [33]. The tolerant line exhibited lower levels of H_2_O_2_ accumulation and lipid peroxidation than the sensitive line. Moreover, this waterlogging-tolerant line showed an earlier upregulation of CAT and SOD activities in response to waterlogging compared to a sensitive one [33].

Despite their toxicity, ROS also function as crucial signaling molecules in plant responses to salt stress and waterlogging [17,34,35,36,37,38,39]. Their rapid production, reactivity, mobility, and tight regulation enable integration with diverse signaling networks. Plasma membrane NADPH oxidases, RESPIRATORY BURST OXIDASE HOMOLOGUEs (RBOHs), are major ROS sources, with several shown to regulate responses to salt and/or waterlogging [40]. In Arabidopsis, RBOHD and RBOHF are well established regulators of ROS-dependent signaling during salt stress, linked to sugar metabolism, proline synthesis, ion homeostasis, Ca^2+^ signaling, hormone pathways, and antioxidant systems [41,42,43,44,45]. These RBOHs are also implicated in waterlogging responses, where ROS signaling intersects with Ca^2+^ and hormone signaling, antioxidant regulation, and processes related to energy metabolism and anaerobic respiration [46,47]. Furthermore, evidence from other plant species supports the central role of RBOH-dependent ROS in responses to salt stress and waterlogging [47,48].

Nitric oxide (NO) is also produced under salt stress and waterlogging [21,49]. NO is generated mainly via oxidative and reductive enzymatic pathways [49,50]. In the oxidative pathway, L-arginine is converted to NO and citrulline by nitric oxide synthase (NOS)-like activity, although the identity of NOS in plants remains unresolved. In the reductive pathway, nitrate reductase (NR) reduces nitrate to nitrite and then to NO, using NADH as an electron donor. Under salt stress, NO synthesis is promoted by upregulating NOS-like or NR activity and by suppressing S-nitrosoglutathione reductase (GSNOR) [49]. The importance of NO in salt stress responses is supported by findings that exogenous NO application alleviates salt-induced damage, whereas scavenging with cPTIO exacerbates injury, underscoring its role in stress tolerance [51]. Functionally, NO contributes to ion homeostasis by modulating ion channel activity, promoting Na^+^ exclusion or sequestration, enhancing osmotic adjustment via osmolyte accumulation (e.g., proline and glycine betaine), and activating antioxidant enzymes [6,40,49,52]. The significance of NO in waterlogging responses has also been highlighted. NO signaling participates in adaptive responses to prolonged waterlogging and is regulated by key transcription factors such as ETHYLENE RESPONSIVE FACTOR (ERF)-VIIs [53,54]. In addition, NO mediates post-translational modifications of respiratory enzymes under hypoxia and modulates RBOH activity, thereby influencing morphological adaptations such as aerenchyma and adventitious root formation [55,56].

Although plant responses to salt stress and waterlogging have been extensively studied individually, these stresses often co-occur and may interact under climate change conditions [57,58]. While ROS and NO are involved in responses to both stresses, combined stress often triggers unique mechanisms not predictable from single-stress responses. In maize, *Suaeda glauca* and *Limnocharis flava*, combined stress exacerbated damage compared to each stress alone [59,60,61]. Conversely, in tomato and *Elaeagnus angustifolia*, salt stress partially mitigated waterlogging damage [58,62]. These findings suggest that responses of plants to a combination of salt stress and waterlogging involve distinct mechanisms. In addition, responses of ROS-scavenging enzyme activities differ between single and combined stresses in tomato, indicating the specificity of ROS-regulatory systems under stress combinations [58]. However, the coordination between ROS and NO regulatory systems under simultaneous salt stress and waterlogging remains largely unknown. This review will discuss the regulatory mechanisms of ROS and NO signaling, their roles in plant responses to salt stress and waterlogging, and, based on previous studies, how plants respond to the combination of these stresses. While plant responses to waterlogging may differ from those to flooding or other hypoxia-related stresses, findings from studies on these stresses are also integrated into the contents.

## 2. Mechanisms Underlying Responses of Plants to Salt Stress Associated with ROS-Regulatory Systems and NO Signaling in Plants

### 2.1. Significance of ROS-Regulatory Systems and ROS Signaling Under Salt Stress

The significance of ROS-scavenging systems in protecting plants against salt stress has been reported in numerous studies. For example, AbdElgawad and co-workers showed that, in maize, increasing salinity in roots and old leaves enhanced total antioxidant capacity, reflected by elevated levels of AA and GSH in roots and increased total tocopherol levels in shoots [63]. They also reported that the activities of CAT and DHAR increased in all organs of salt-stressed plants, while SOD, APX, GR, and glutathione-S-transferase (GST) activities rose specifically in the roots. Genetic evidence further supports the crucial role of ROS-scavenging systems in the protection of plants against salt stress. It was demonstrated that overexpression of APX7B from durum wheat enhanced Arabidopsis tolerance to multiple abiotic stresses, including salt stress [64]. This improved tolerance was associated with increased activities of ROS-scavenging enzymes such as CAT, SOD, and POD. In addition, expression of the *Catalase 1* gene from wheat in *E. coli* and yeast conferred enhanced salt stress tolerance in these organisms [65,66]. Collectively, these results suggest that while specific ROS-scavenging systems may act in a tissue- or organ-dependent manner, their roles in salt stress responses appear to be conserved across diverse organisms. Exogenous application of bio-stimulants has also been recognized as a promising approach to enhance salt stress tolerance, with many positive effects linked to ROS-scavenging capacity. For example, exogenous chitosan, tyrosine, ZnO nanoparticles, and methyl jasmonate (MJ) enhanced the tolerance of rapeseed, kale, maize, and walnut, respectively, through the activation of non-enzymatic and/or enzymatic antioxidant systems [67,68,69,70]. Of these studies, enhanced salt stress tolerance and ROS-scavenging capacity were accompanied by the maintenance of photosynthetic activities in rapeseeds, maize, and walnut [67,68,70]. Chloroplasts are highly sensitive to oxidative damage and represent a major source of ROS under abiotic stresses, including salt stress [11]. Elevated Na^+^ limits water uptake, causing osmotic stress and stomatal closure that reduce CO_2_ availability and lead to excess reducing power, resulting in oxidative damage to photosystem II reaction center proteins [9,10]. Therefore, protecting chloroplast function, especially photosystem II, from oxidative damage likely represents an effective strategy to confer salt stress tolerance in plants.

**Figure 1:**
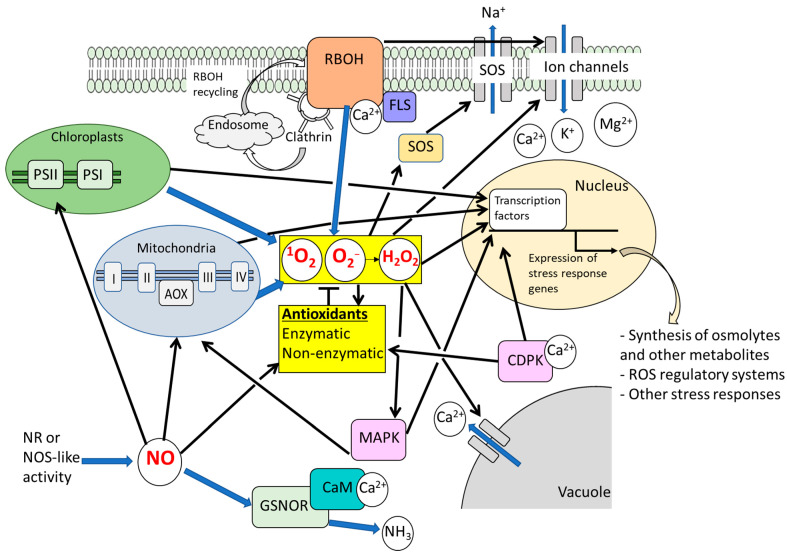
ROS- and NO-dependent signaling in plant responses to salt stress. ROS produced by RBOHs are integrated with other signaling pathways involving Ca^2+^, MAPKs, and CDPKs. RBOH-derived ROS also regulate SOS-dependent mechanisms that maintain ion homeostasis under salinity. NO modulates ROS levels by activating antioxidant systems and influencing RBOH activity. Excessive ROS generated in chloroplasts and mitochondria can damage these organelles. In particular, protecting chloroplast functions from oxidative damage is crucial for plant tolerance to salt stress. However, ROS produced in these organelles may act as regulators of intracellular communication, including retrograde signaling from chloroplasts and mitochondria to the nucleus.

Although ROS generated by salt stress can impair plant growth and development, ROS produced in different cellular compartments can also function as signaling molecules that regulate protective mechanisms against salt-induced damage [71] (Figure 1). While the studies using bio-stimulants described above indicate that preventing oxidative damage to chloroplasts and photosynthetic apparatus is critical for salt tolerance, contrasting evidence suggests that exogenous H_2_O_2_ itself can also exert positive effects on salt tolerance and photosynthetic function. In wheat and maize, it was demonstrated that seed priming with H_2_O_2_ prevented salt stress-induced thylakoid stacking, thereby avoiding excess energy accumulation and enhancing photosynthetic activity under salt stress [72,73]. These findings indicate that the level of ROS as signaling molecules must be tightly regulated to protect chloroplasts under salt stress. The candidate of ROS-related signals associated with chloroplasts, which contribute to salt stress responses, can be retrograde signaling. It is well established that signals from chloroplasts, known as retrograde signaling, regulate the expression of nuclear genes under abiotic stresses [74], and ROS are recognized as important regulators of these signals [74]. Although the role of ROS-dependent retrograde signaling in salt stress responses remains poorly understood, chloroplast retrograde signaling involving chlorophyll precursors has been implicated in the salt response of the marine alga *Dunaliella salina*, a species with exceptional tolerance to abiotic stresses, particularly hypersaline conditions [75]. Further work is needed to clarify how retrograde signaling contributes to salt stress regulation in higher plants. Beyond photosynthesis, H_2_O_2_ priming enhances additional processes relevant to salt tolerance. In wheat, priming improved growth, increased pigments, proline, and mineral uptake (K^+^, Ca^2+^, Mg^2+^), while reducing Na^+^ accumulation [76]. It also altered metabolites such as glucose, arabitol, tyrosine, and asparagine, which may promote osmotic adjustment and antioxidant defense [72]. Collectively, these findings underscore the dual role of ROS in salt stress as damaging agents and as signaling molecules that trigger acclimatory responses.

ROS act as signaling molecules under salt stress and are produced in part through the activity of NADPH oxidases, known as RESPIRATORY BURST OXIDASE HOMOLOGUEs (RBOHs) [30,77]. In Arabidopsis, the importance of RBOHD and RBOHF in regulating salt tolerance has been demonstrated by the strong sensitivity of double mutants deficient in both genes [43]. Several studies have elucidated RBOHD-mediated salt stress responses. Trafficking of plasma membrane-localized RBOHD to endosomes, followed by its recycling back to the plasma membrane, is essential for ROS production under salt stress [78]. Mutants lacking ENDOMEMBRANE-TYPE CA-ATPASE 4 (ECA4), which is required for recycling proteins from endosomes to the plasma membrane, as well as mutants deficient in CLATHRIN LIGHT CHAIN 2 (CLC2) and CLATHRIN HEAVY CHAIN 2 (CHC2), which are required for endocytosis, show impaired ROS production in response to salinity. In addition, plants lacking ECA4 display reduced accumulation of RBOHD at the plasma membrane under salt stress. These findings indicate that endocytosis is essential for RBOHD-dependent ROS signaling under salinity. Downstream signaling events mediated by RBOHD have also been uncovered. For example, RBOHD, together with FLAGELLIN SENSITIVE 2 (FLS2), regulates the expression of a gene encoding PHYTOCHROME INTERACTING FACTOR 4 (PIF4) under salt stress [43]. This pathway is linked to the modulation of metabolites such as aspartic acid, L-proline, D-ribose, and indoleacetaldehyde [44]. Furthermore, the involvement of RBOHF in salt stress responses has been demonstrated. Melatonin, recently recognized as an important inducer of tolerance to abiotic stresses, including salinity, rapidly stimulates *RbohF* transcription and ROS production. Both responses are largely abolished in mutants deficient in RBOHF, providing genetic evidence for RBOHF involvement [45]. Moreover, the regulator of the RBOH-dependent mechanism has been identified in soybean. Salt-induced H_2_O_2_ modifies the transcription factor NAC WITH TRANS-MEMBRANE MOTIF1-LIKE 1 (NTL1), promoting its nuclear translocation and activation of transcript encoding RBOHB expression, thereby amplifying ROS signaling [77]. These observations highlight the essential role of RBOH-dependent ROS signaling in plant salt stress responses. However, not all RBOHs are involved, suggesting specificity in the signaling pathways they mediate. Supporting this, expression of RBOHA and RBOHI in rice is upregulated by salt stress, showing a distinct expression pattern compared to other abiotic stresses [79].

In Arabidopsis, RBOHD also regulates long-distance signaling via the so-called “ROS wave” [80], activated under salt stress together with the vacuolar ion channel TWO PORE CHANNEL1 (TPC1), a central regulator of Ca^2+^ waves [81]. This reflects the tight interplay between ROS and Ca^2+^ signaling. Indeed, Ca^2+^ binding to the EF-hand motif of RBOH proteins is required for their activity [82], and RBOHs were shown to modulate Ca^2+^ influx via regulating Ca^2+^ channels [80,81], suggesting that RBOHs might be one of the key mediators that link ROS and Ca^2+^ signaling. Other studies also demonstrated the integration of Ca^2+^ and ROS signaling. For example, CALCIUM-DEPENDENT PROTEIN KINASES (CDPK7) activate stress-responsive genes involved in ROS scavenging, enhancing tolerance of rice to salt stress [83]. Ca^2+^ signals also enhance ROS-scavenging enzymes and secondary metabolites, such as phenolics and flavonoids, which mitigate oxidative damage [84]. In addition, Ca^2+^ activates these acclimatory systems through transcription factors including WRKY, MYELOBLASTOSIS (MYB), NAM,-ATAF1/2-CUC2 (NAC), and APETARA2/ERF (AP2/ERF) families [48].

ROS also integrate with MITOGEN-ACTIVATED PROTEIN KINASE (MAPK) signaling cascades during salt stress. In Arabidopsis, HEAT SHOCK TRANSCRIPTION FACTOR A4A (HSFA4A) mediates salt stress tolerance via regulation of MAPK3 and 6 as well as pathways associated with oxidative stress responses [85]. More recently, MAPK3 and 6 were shown to modulate ROS-scavenging enzyme activity through MAPK9 under salt stress [86]. This pathway also connects to ethylene signaling, mitochondrial respiration, and mitochondrial ROS-regulatory systems involving ALTERNATIVE OXIDASEs (AOXs) [86]. While these positive pathways enhance salt tolerance, negative signaling mechanisms also exist. For example, Li and co-workers described a SIT1 (SALT INTOLERANCE 1, a receptor-like kinase)-MAPK3/6 cascade that increases salt sensitivity by altering ROS and ethylene homeostasis [87]. In addition, the integration of MAPK-dependent signals with CDPKs has also been suggested in several studies. Thus, ROS-regulatory systems, in concert with other pathways involving MAPKs, might fine-tune responses depending on stress intensity and duration. Consistent with this, effects of salt stress on mitochondrial respiration vary with NaCl concentration [86].

Taken together, these findings suggest that cellular ROS levels are tightly regulated through the integration of multiple processes, including Ca^2+^ signaling, kinase cascades, endocytosis, and transcriptional networks.

### 2.2. Significance of NO Signaling Under Salt Stress

Nitric oxide (NO) has emerged as a key regulator of plant responses to salt stress. Previous studies demonstrated its ability to mitigate salinity-induced damage by activating ROS-scavenging systems in multiple species. In Arabidopsis and chickpea, NO treatment enhanced the activities of SOD, APX, and CAT, while reducing mitochondrial release of H_2_O_2_ and O_2_^−^, and MDA accumulation [6,88]. In barley, exogenous NO increased non-enzymatic antioxidants such as GSH and ASA, together with SOD, CAT, APX, and GR activities, thereby improving membrane stability and photosynthetic performance under salt stress [89]. Similarly, sodium nitroprusside (SNP) treatment activated the ascorbate–glutathione cycle in salt-stressed *Nitraria tangutorum* [51]. By contrast, the inhibition of NO signaling with the scavenger cPTIO suppressed antioxidant enzymes (SOD, APX, GR, DHAR, and MDHAR), reduced GSH/GSSG ratios, and caused excess ROS accumulation and membrane damage [90]. These findings highlight NO as a crucial signaling molecule enhancing ROS-scavenging capacity under salinity. In addition, Ca^2+^/Calmodulin (CaM) complexes directly interact with GSNOR and inhibit its activity, promoting NO accumulation and ion homeostasis to enhance salt tolerance [91]. Together, these findings suggest that integration of Ca^2+^ and NO signaling plays a crucial role in maintaining optimal ROS levels under salt stress.

Given their roles in regulating ROS homeostasis, NO signals are likely to interact with processes operated in ROS-producing organelles such as chloroplasts and mitochondria. Several studies confirm the ability of NO to alleviate salt-induced damage to physiological processes [92]. For example, SNP treatment improved growth and photosynthetic CO_2_ assimilation in lettuce [92]. In wheat, NO application to seeds or seedlings increased photosynthetic pigment content and efficiency, enhanced accumulation of osmolyte, soluble sugar, and protein, and improved nutrient uptake, while limiting Na^+^ accumulation [88,89]. Similar protective effects on photosynthesis and chloroplast structure were reported in chickpea, cotton, and *Brassica juncea* [6,93,94]. The maintenance of photosynthetic activity may be associated with the protection of reaction center proteins in PSII. It was demonstrated that NO application attenuated the negative impact of salt stress on the PSII reaction center in egg plants [10]. In addition, the accumulation of Calvin–Benson cycle enzymes, including RUBISCO and RUBISCO ACTIVASE, was also shown to be maintained by exogenous application of SNP in mangrove plant *Avicennia marina* [95]. These findings suggest that NO supports both thylakoid and stromal functions under salt stress. NO also directly reacts with ·O_2_^−^ generated from the mitochondrial electron transport chain, forming peroxynitrite (ONOO^−^). This reaction may regulate free NO levels [96] and points to a role for NO in modulating mitochondrial activity during salt stress. In wheat, NO seed priming enhanced respiration, ATP synthesis, and amylase activity under salt conditions [88]. Collectively, these findings indicate that NO promotes energy homeostasis by sustaining chloroplast and mitochondrial functions, while also reinforcing osmotic adjustment and nutrient metabolism. This role is further supported by evidence that NO regulates sucrose transporter genes, thereby supplying energy and structural components for young leaf growth [97].

Taken together, these findings highlight the strong connections between ROS-regulatory systems and NO signaling. Both ROS and NO govern a wide range of shared cellular processes [98], and their interplay represents a central mechanism of salt stress tolerance. Under salinity, both molecules are produced with comparable kinetics and often act synergistically to regulate seed germination, growth, physiology, antioxidant defense, and ion balance [16,71,99,100,101,102]. In tomato roots subjected to salt stress, in situ fluorescent staining confirmed the simultaneous accumulation of ROS and NO [103]. Likewise, proteomic analysis of citrus plants revealed overlapping functions of H_2_O_2_ and NO during acclimation, with priming by either molecule reprogramming the expression of more than 50 proteins, nearly half of which were associated with photosynthetic processes such as the RUBISCO large subunit, RUBISCO ACTIVASEs, and FRUCTOSE-1,6-BISPHOSPHATE ALDOLASE [99,103,104]. These findings underscore the integrated roles of NO and ROS as co-regulators of plant metabolism and stress adaptation. In addition, salt stress elicits specific responses that can be broadly divided into two phases: a rapid osmotic effect, followed by a slower ionic toxicity effect [7,8,13,105]. For each phase, plants deploy distinct adaptive strategies, as exemplified below.

### 2.3. Roles of ROS and NO Signals in Alleviation of Osmotic Effects Caused by Salt Stress

Under osmotic stress conditions, imposed by drought or salinity, plants activate various signaling events involving abscisic acid (ABA), ROS, and Ca^2+^. Among the known plant hormones, ABA is well recognized for its crucial role in mediating salt response signals to regulate plant growth adaptation [106,107]. The primary response to osmotic stress is stomatal closure, mediated by ROS- and Ca^2+^-dependent signaling together with enhanced ABA production in guard cells, which reduces transpiration and water loss [108,109]. Indeed, stomatal guard cells represent a unique plant cell model that can serve as an ideal model for the analysis of stimulus–response signaling tracks in higher plants [110]. The process of stomatal opening and closure represents a key response that adjusts the plant metabolism under continuously changing environmental conditions. Stomata manage the balance between the CO_2_ entry into leaves for photosynthesis and the transpirational water streams that adjust the plant temperature and carry various substances throughout the plant body. Thus, stomatal control is crucial for plant growth and development under normal and stress conditions, including not only drought and high salinity, but also low and high temperatures, pathogens, light signals, and phytohormones such as ABA, jasmonic acid (JA), salicylic acid (SA), and ethylene, that affect stomatal aperture [98,111,112].

**Figure 2:**
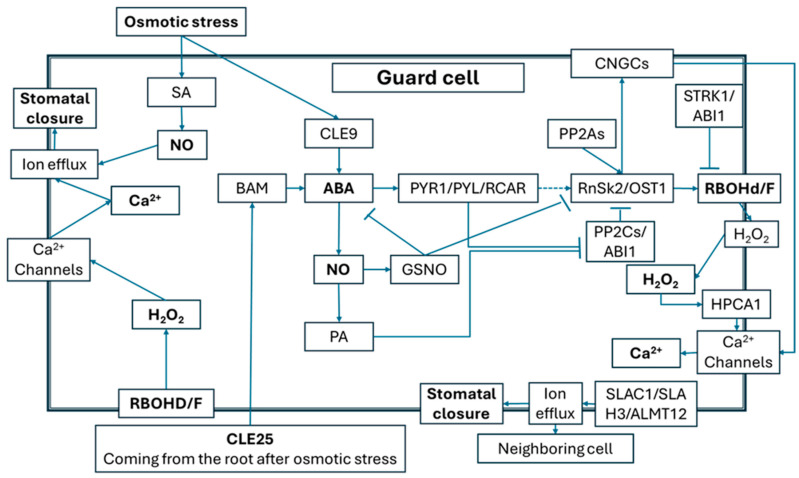
Salt-induced osmotic stress is perceived primarily by the roots, and the resulting signal is transmitted to the leaves. In the leaves, ABA signaling activates RBOH-dependent ROS production, which in turn regulates ion channel activity in guard cells, adjusting their osmotic pressure and leading to stomatal closure. NO and other plant hormones further modulate these signaling pathways, fine-tuning the stomatal response under salt stress.

We provide an overview of the stomatal closure signaling pathway, a key plant response to drought and salinity, with emphasis on ROS and NO (Figure 2). In Arabidopsis, the mobile peptide CLAVATA3/EMBRYO SURROUNDING REGION 25 (CLE25), produced in root cells sensing osmotic stress, transmits signals to leaves via vascular tissues. BARELY ANY MERISTEM (BAM) receptors in leaves decode this signal as elevated ABA in guard cells [113]. ABA activates its receptors (PYR1/PYL/RCAR), which inhibit PROTEIN PHOSPHATASE 2Cs (PP2Cs), allowing phosphorylation of SNF1-RELATED PROTEIN KINASE 2 (SnRK2) and activation of downstream ABA-responsive factors such as ABFs [114]. SnRK2s (SnRK2.2, 2.3, 2.6/OPEN STOMATA (OST) 1, 2.8) also interact with PP2A subunits during ABA-induced stomatal closure [115]. ABA signaling promotes ROS production in guard cells [116,117]. RBOHD and RBOHF generate H_2_O_2_ required for ABA–Ca^2+^ signaling [116,118]. RBOHF activity is regulated by OST1 and phosphatidic acid [117], and balanced by SALT TOLERANCE RECEPTOR-LIKE CYTOPLASMIC KINASE 1 (STRK1) and ABSCISIC ACID-INSENSITIVE 1 (ABI1) to prevent toxic H_2_O_2_ overproduction [28,119]. Controlled H_2_O_2_ activates the membrane sensor HYDROGEN PEROXIDE-INDUCED CALCIUM-INCREASING ACTIVATOR 1 (HPCA1), which triggers Ca^2+^ influx [120]. Increased cytosolic Ca^2+^ then activates anion channels, SLOW ANION CHANNEL 1 (SLAC1), SLAC1 HOMOLOGUE 3 (SLAH3), aluminum-activated malate transporter 12 (ALMT12), driving K^+^ efflux, water loss, and guard cell shrinkage, ultimately closing stomata [121,122,123,124]. This CLE25–BAM–ABA–SnRK2/OST1–RBOHD/F–H_2_O_2_–HPCA1–Ca^2+^–anion channel pathway, with regulation by STRK1 and ABI1, thus reduces transpiration under osmotic stress. Ca^2+^ also reinforces ROS production: CALCINEURIN B-LIKE–CBL-INTERACTING PROTEIN KINASE (CBL–CIPK) complexes directly phosphorylate RBOHF, while CBLs with CIPK11/26 and OST1 further enhance its activation [119,125]. Other branches include CLE9, expressed in guard cells, which signals via ABA, OST1, RBOH, H_2_O_2_/NO, and SLAC1 [126]. In addition, a ROS-independent ABA–SnRK2/OST1–Ca^2+^ module involves CYCLIC NUCLEOTIDE-GATED CHANNELs (CNGC), which are phosphorylated by OST1 to promote Ca^2+^ influx and stomatal closure [127,128]. NO plays both positive and negative roles in stomatal regulation. It supports ABA-induced closure by inactivating inward K^+^ channels via GDP-MANNOSE PYROPHOSPHORYLASE/ADP-ribose (GMP/ADPR)-mediated Ca^2+^ increases [129], by inducing phosphatidic acid (PA) production and Ca^2+^ release, and by activating RBOHD/F through PA binding [117,130]. PA also inhibits ABI1 and activates SnRK2.4/2.10 and PP2A [130]. Conversely, NO exerts feedback inhibition by S-nitrosylating OST1, suppressing its kinase activity, and by promoting GSNO accumulation, which disrupts ABA signaling [130,131]. This negative regulation likely balances water saving with gas exchange. Finally, NO mediates SA-induced stomatal closure under salt stress. Exogenous SA induced both NO production and closure, effects abolished by NO scavengers, inhibitors, or in nitrate reductase mutants (nia1, nia2, nia1/nia2), confirming the role of NR-dependent NO in this process [132].

Production of osmolytes is another key strategy for mitigating the effects of salt-induced osmotic stress in plants, and their links with ROS and NO signaling have been reported. By scavenging harmful ROS and preserving antioxidative enzymes, osmolytes strengthen the antioxidant defense system [133,134]. Amino acid-derived osmoprotectants such as proline, arginine, alanine, leucine, glycine, serine, valine, and γ-aminobutyric acid (GABA) accumulate under salinity, modulating the osmotic potential of cells to facilitate water uptake. They also stabilize proteins and membranes and act as nitrogen reservoirs [135,136]. Among these, proline is one of the most extensively studied osmolytes. In the glutamate pathway, proline is synthesized from glutamate via the intermediate Δ^1^-pyrroline-5-carboxylate (P5C), catalyzed by Δ^1^-PYRROLINE-5-CARBOXYLATE SYNTHETASE (P5CS) and Δ^1^-PYRROLINE-5-CARBOXYLATE REDUCTASE (P5CR) [137]. Alternatively, in the ornithine pathway, proline is produced from ornithine via Orn-δ-AMINOTRANSFERASE (δ-OAT) [138]. Constitutive expression of the Oat gene in rice seedlings enhances δ-OAT activity, antioxidant capacity, and tolerance to drought and osmotic stress [139]. However, under salt stress, the glutamate pathway is preferentially utilized due to increased P5CS expression, underscoring its pivotal role in proline accumulation during osmotic adjustment [140]. It should be noted that these enzymes involved in proline metabolism function in chloroplasts and mitochondria [141], suggesting that modulation of proline metabolism might be linked with the maintenance of photosynthesis and respiration [141].

Sugar alcohols are also well-recognized osmolytes involved in plant responses to salt stress. Beyond their osmotic roles, compounds such as mannitol, myo-inositol, and sorbitol function as regulators of ROS and molecular chaperones [142,143]. Enhanced salt tolerance through overexpression of the bacterial mannitol-producing gene mtlD has been reported in several plant species [141]. The mtlD-mediated tolerance was associated with increased antioxidant enzyme activity and reduced H_2_O_2_ and MDA accumulation [144]. Inositol and its derivatives contribute to salt tolerance by protecting cellular structures from ROS damage while maintaining cell turgor [141]. Overexpression of a gene encoding MYO-INOSITOL-1-PHOSPHATE SYNTHASE (MIPS) in sweet potato conferred enhanced tolerance, not only to salt stress, but also to drought and stem nematodes [145]. These transgenic plants additionally exhibited upregulation of genes involved in inositol biosynthesis, phosphatidylinositol and ABA signaling, photosynthesis, and ROS scavenging [145]. Sorbitol is also considered an important osmolyte under salt stress. A recent study demonstrated that overexpression of the transcription factor G-BOX BINDING FACTOR 3 (GBF3) in pear enhanced salt tolerance in pear calli and in Arabidopsis [146]. GBF3 binds to the promoters of genes encoding SORBITOL DEHYDROGENASE 1 (SDH1) and AGPase LARGE SUBUNIT 2 (APL2), key enzymes in the sorbitol pathway and starch synthesis, respectively. Activation of this pathway enhanced salt tolerance by increasing AGPase activity, soluble sugar content, and SDH activity, thereby improving ROS scavenging and ion homeostasis. These findings suggest that pathways involving sugar alcohols regulate tolerance of plants to salt stress via modulating various signaling.

Glycine betaine (GB) is another well-studied osmolyte with roles in stress responses. Although its integration with ROS and NO signaling is not fully established, several studies support such a connection. Upregulation of the BETAINE ALDEHYDE DEHYDROGENASE (BADH) gene has been proposed as a biomarker in salt-stressed wheat [147]. Likewise, Arabidopsis plants expressing a novel BADH gene (ScBADH) accumulated SOD, proline, and GB under salinity [148]. Notably, GB accumulates mainly in chloroplasts [134], suggesting an essential role in maintaining photosynthetic machinery and associated processes.

### 2.4. ROS/NO-Related Signaling Events Mediating Plant Responses to Salt Stress-Induced Ionic Toxicity

Maintenance of ion homeostasis is another key process that protects plant cells against salt stress. The SALT OVERLAY SENSITIVE (SOS) pathway (SOS1, SOS2, and SOS3) is the most established signaling system counteracting Na^+^ toxicity in plants [7,8,13,106]. When roots sense high external Na^+^, membrane-bound Ca^2+^ channels open, elevating cytosolic Ca^2+^ levels. This signal is decoded by CBL–CIPK complexes, which activate SOS2 and SOS3. Together, they stimulate SOS1, a plasma membrane Na^+^/H^+^ antiporter that extrudes Na^+^ into the apoplast or soil [149,150]. Loss-of-function mutants of SOS2 in Arabidopsis show hypersensitivity to Na^+^ but not to osmotic stress, confirming the specificity of this pathway [151].

Although the potential roles of ROS and NO in the SOS pathway remain poorly understood, limited evidence suggests some involvement, for example, SOS2 interacts with NUCLEOSIDE DIPHOSPHATE KINASE 2 (NDPK2) and CAT, linking Na^+^ signaling to H_2_O_2_ metabolism [152]. In addition, SOS1 mRNA is normally unstable (half-life ~10 min), but its stability under salt stress is enhanced by H_2_O_2_, likely via NADPH oxidase-derived ROS [153]. These reports hint at a connection between ROS and the SOS system but leave a clear gap requiring further study. Furthermore, involvement of NO in the regulation of SOS signaling was proposed by the finding that NO promotes Ca^2+^-SOS signaling, leading to efflux of Na^+^ under salt stress in Glycyrrhiza uralensis [49,154]. Several CNGCs have been implicated in abiotic stress responses, including salt stress [48]. In rice, CNGC14, a homolog of Arabidopsis CNGC10, regulates Na^+^ and K^+^ transport; mutations in CNGC14 increase salt sensitivity by elevating Na^+^ accumulation and disrupting the K^+^/Na^+^ ratio [48]. Functions of CNGC2 in regulating heat and highlighting stress responses were also associated with ROS signaling [155,156]. However, the integration of CNGC activity with ROS and NO signaling under salt stress remains to be elucidated.

Beyond SOS signaling, ROS also play essential roles in Na^+^/K^+^ homeostasis. NADPH oxidases RBOHD and RBOHF regulate ion balance in Arabidopsis roots [41,42]. Double mutants deficient in both RBOHD and RBOHF exhibit hypersensitivity to salt stress, characterized by elevated Na^+^, reduced K^+^, and higher Na^+^/K^+^ ratios [41]. These effects are partially alleviated by exogenous H_2_O_2_ treatment, confirming the role of RBOH-dependent H_2_O_2_ in salt responses and ion homeostasis. In addition, the deficiency in RBOHF resulted in increased ROS accumulation in vascular cells, leading to accumulation of excess Na^+^ in xylem sap, which causes shoot hypersensitivity to salinity in the shoot [42]. This RBOHF-dependent process was shown to be associated with ethylene signaling. Salt stress induces ethylene, which activates RBOHF to reduce root Na^+^ influx and xylem loading, while a parallel ethylene-dependent, RBOHF-independent pathway enhances K^+^ uptake via HIGH AFFINITY K+ TRANSPORTER 5 (HAK5) [112]. Supporting evidence from grafted cucumber demonstrated that pumpkin rootstocks enhance cucumber salt tolerance, accompanied by reduced leaf Na^+^ content and increased root Na^+^ exclusion via Na^+^/H^+^ antiporter activity, along with enhanced expression of transcripts encoding RBOHs, SOS1, and H^+^-ATPase [157]. These findings suggest that RBOH-dependent ROS signaling regulates ion transport and may mediate root-to-shoot communication under salt stress, warranting further investigation into the tissue-specific roles of different RBOHs. Integration of NO signaling with ion homeostasis has also been reported. Application of the NO donor SNP increased endogenous NO and promoted selective transport of K^+^ and Na^+^, maintaining K^+^/Na^+^ homeostasis in Kandelia obovata, pak choi, wheat, rice, and soybean [52,101,158,159,160]. The importance of NO was further supported by the genetic evidence that Arabidopsis noa1 mutants, deficient in NO synthesis, accumulate more Na^+^ and less K^+^ in shoots under NaCl stress [161]. This maintenance of ion balance may be at least partly due to NO-mediated enhancement of plasma membrane H^+^-ATPase activity, which provides the driving force for K^+^ uptake and Na^+^ efflux under salt stress [49].

## 3. Mechanisms Underlying Responses of Plants to Waterlogging Associated with ROS-Regulatory Systems and NO Signaling

### 3.1. Significance of ROS-Regulatory Systems and ROS Signaling Under Waterlogging

Waterlogging disrupts root oxygen supply, resulting in oxidative stress characterized by excessive ROS accumulation [21,31]. To cope with this stress, plants activate synergistic antioxidant defenses, in which enzymatic antioxidants and non-enzymatic compounds act in concert with ROS–NO signaling [162,163]. Flooding or hypoxia induces the upregulation of genes encoding APX, CAT, and enzymes involved in GSH synthesis in barley, soybean, and mung bean [31]. Waterlogging also modulates AOX expression and enhances antioxidant enzyme activities [32]. Secondary metabolites such as flavonoids, carotenoids, and phenolics further function as ROS scavengers, membrane stabilizers, and regulators of oxidative signaling [164]. The significance of ROS-scavenging systems was also underscored by cultivar comparisons. In sesame, tolerant genotypes exhibit higher SOD and peroxidase activities and lower MDA levels than sensitive ones [33]. Furthermore, the activation of these systems is closely linked with hormone signaling. Ethylene, JA, and ABA positively regulate ROS signaling, which contributes to stomatal regulation and integrates with transcription factor functions controlling ROS-scavenging gene expression [31].

Under waterlogging and hypoxia, impaired mitochondrial electron transport leads to electron leakage from complexes I and III, causing ROS accumulation, particularly ·O_2_^−^ and H_2_O_2_ [22]. Thus, controlling ROS accumulation through mitochondrial regulation and adjustments in respiration-related metabolism represents a critical process under waterlogging. Recent studies have shown that, in addition to enhancing ROS scavenging, plants suppress the mitochondrial electron transport chain by lactylating key mitochondrial acetyl-CoA providers, thereby reducing ROS production [165]. In addition to respiration, photosynthesis is also known as a process that is sensitive to waterlogging. Waterlogging restricts CO_2_ and O_2_ diffusion in roots and stems, leading to photosynthetic damage [23]. A comparison of two tomato cultivars differing in waterlogging sensitivity revealed that sensitive genotypes exhibit stomatal photosynthetic limitations, whereas tolerant ones experience non-stomatal impairments such as chloroplast dysfunction [166]. Furthermore, waterlogging was shown to cause severe reductions in photosynthetic activity and pigment levels, accompanied by increased H_2_O_2_ and MDA accumulation, linking oxidative stress with photosynthetic disruption. Collectively, these findings suggest that protecting organelle functions essential for energy homeostasis from oxidative damage is critical for plant responses to waterlogging.

The crucial roles of ERF-VII transcription factors in hypoxia perception and the regulation of multiple processes have been highlighted in previous studies. Overexpression of ERF-VIIs enhances the expression of genes encoding anaerobic respiration enzymes, such as ALCOHOL DEHYDROGENASE (ADH) and PYRUVATE DECARBOXYLASE (PDC), thereby maintaining energy homeostasis and improving waterlogging tolerance [167,168]. In wheat, ERF-VII.1 was shown to induce genes involved in both anaerobic respiration and ROS-scavenging systems, functioning together with the negative regulator SANT DOMAIN TRANSCRIPTION FACTOR 18.1 (SAB18.1). Under normal conditions, SAB18.1 suppresses hypoxia responses, whereas rapid activation of ERF-VII.1 disrupts this inhibition [169]. In maize, EREB180, a member of ERF-VII, activates ROS-scavenging enzymes, while EREB179 functions as its negative regulator [170,171].

**Figure 3:**
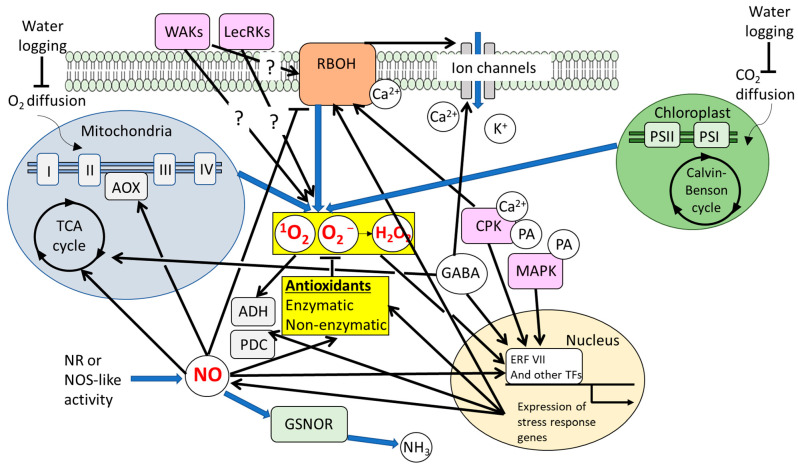
Waterlogging restricts the diffusion of O_2_ and CO_2_, thereby inhibiting respiration and photosynthesis through disruptions in mitochondrial electron transport and the Calvin–Benson cycle. Similar to salt stress signaling, ROS produced by RBOHs are integrated with pathways involving Ca^2+^, MAPKs, and CDPKs, which activate antioxidant systems and other stress responses such as ERF-VII-mediated signaling, anaerobic respiration, and ion homeostasis. Additionally, signals derived from the cell wall and phosphatidic acid (PA) are implicated in the regulation of ROS-dependent signaling. NO also modulates these ROS-mediated pathways.

ROS act as double-edged molecules, capable of causing cellular damage or activating protective signaling, thereby playing a central role in acclimation to waterlogging [18,20,24,172] (Figure 3). Cell-wall-based sensors such as WALL-ASSOCIATED KINASEs (WAKs) and LECTIN RECEPTOR KINASEs (LecRKs) have been shown to modulate ROS-dependent signaling under waterlogging [23]. RBOH-mediated ROS signaling is also implicated in the regulation of energy homeostasis. In Arabidopsis, double mutants deficient in RBOHD and RBOHF display enhanced sensitivity to hypoxia, accompanied by reduced expression of ADH1 and PDC1 transcripts and suppressed activities of ADH, PDC, and lactate dehydrogenase [46]. In addition, ATP synthesis under hypoxia is impaired in both single and double mutants deficient in RBOHD and/or RBOHF, with the most severe effects in the double mutant. RBOHD-dependent stress responses during waterlogging also require the RHO-LIKE SMALL G PROTEIN 2 (ROP2), further highlighting the complexity of ROS signaling pathways [173].

Disruption of ROS production as a signaling molecule, caused by deficiencies in RBOHD and/or RBOHF, is accompanied by impaired Ca^2+^ elevation [46], highlighting the tight link between ROS and Ca^2+^ signaling under waterlogging. ROS and Ca^2+^ waves act together to trigger rapid systemic signals that connect hypoxic roots with shoots [80], intersecting with ethylene, ABA, and auxin pathways to regulate adaptive traits such as aerenchyma and adventitious root formation [174,175]. In maize, Ca^2+^ signaling was shown to regulate ADH activity, which is also controlled by RBOH-dependent ROS, thereby enhancing tolerance to hypoxia [176]. Furthermore, hypoxia-induced gamma-aminobutyric acid (GABA) accumulation plays a critical role in restoring membrane potential and preventing ROS-induced disruption of cytosolic K^+^ homeostasis and Ca^2+^ signaling. Elevated GABA levels may restore membrane potential via pH-dependent regulation of H^+^-ATPase and/or increased energy generation through activation of the GABA shunt and TCA cycle [177]. This GABA-dependent mechanism is also linked to transcriptional upregulation of transcripts encoding RBOHs. Ca^2+^-dependent signaling is further integrated with lipid and kinase pathways. PA binds to CDPK12, promoting its nuclear translocation and phosphorylation of ERF-VIIs in Arabidopsis [178]. PA also interacts with MAPK3 and MAPK6 [179], which phosphorylate RELATED TO AP2.12 (RAP2.12), a master transcription factor in hypoxia signaling, thereby modulating its activity.

It was shown that waterlogging induces RBOH-dependent ROS production, which activates autophagy [32]. In mutants deficient in RBOHs, upregulation of autophagosomes was less pronounced than in the wild type upon waterlogging. However, the accumulation of ROS and the level of cell death in the roots of atg mutants that are deficient in autophagy-related genes were higher than those in the wild type after waterlogging. These results suggest that autophagy induced during waterlogging mitigates programmed cell death (PCD) [32].

Taken together, these findings indicate that ROS and Ca^2+^ signaling are integrated with various pathways associated with ion transport, energy homeostasis, and kinases.

### 3.2. Significance of NO Signaling Under Waterlogging

The significance of NO in the regulation of waterlogging responses of plants has also been demonstrated in many studies. The effects of NO under waterlogging depend on concentration, antioxidant capacity, and exposure duration [24,31]. The above-mentioned signals involving anaerobic respiration, PA, and MAPKs are considered as early signals in response to hypoxia [31]. In contrast, NO-dependent signaling contributes to late responses to hypoxia [31]. During the initial phase of hypoxia, gradual ethylene accumulation induces the expression of the phytoglobin gene encoding HEMOGLOBIN 1 (HB1), which reduces NO levels and thereby stabilizes ERF-VIIs [180,181]. By contrast, under prolonged hypoxia, stabilized ERF-VIIs promote NO accumulation [53,54].

Nitrate treatment has been shown to elevate root NO levels in cucumber, leading to reduced H_2_O_2_ and MDA accumulation and highlighting the role of NO signaling in alleviating oxidative stress under waterlogging [182]. NO also regulates antioxidant activity through post-translational modifications such as S-nitrosylation and tyrosine nitration, which can either enhance or suppress enzyme function [183,184]. Together with transcriptional regulation, these modifications underpin plant stress adaptation to waterlogging [17,184]. For instance, NO-induced S-nitrosylation inhibits ACONITASE, leading to the accumulation of intermediates such as citrate, which induces ALTERNATIVE OXIDASE 1A (AOX1A) expression and enhances its activity in Arabidopsis [54,185]. In contrast, NO bursts can inhibit COX activity [54]. This differential modulation of AOX and COX suggests that NO plays a key role in mitochondrial respiration under hypoxia [54].

Beyond influencing antioxidant capacity, NO also targets ROS-producing RBOHs through post-translational modifications. Specifically, NO attenuates RBOHD activity via S-nitrosylation at Cys890, functioning as a negative feedback mechanism [186]. Conversely, ROS can inactivate GSNO reductase (GSNOR), thereby altering S-nitrosothiol pools and enhancing NO bioactivity [187]. This reciprocal regulation ensures effective ROS–NO signaling. The ROS–NO axis was shown to be tightly interconnected with hormone signaling. Fine-tuned coordination between ethylene signaling and NO accumulation is crucial for appropriate plant responses to waterlogging [188]. Under low oxygen and limited NO, ERF-VIIs are stabilized through N-terminal cysteine oxidation by plant cysteine oxidases, thereby inducing hypoxia-responsive genes [189]. Ethylene further enhances phytoglobin-mediated NO scavenging, pre-adapting plants before hypoxia intensifies [180]. Upon reoxygenation, a ROS burst occurs, shifting ROS–NO interactions toward repair and recovery. While balanced redox signaling promotes acclimation, imbalances can trigger programmed cell death [21,181]. ERF-VIIs, redox enzymes, and ROS-scavenging systems coordinate these transitions by integrating ROS–NO sensing, dynamic NO production and scavenging, and ROS-mediated local and systemic signaling, thereby fine-tuning hypoxia tolerance, morphological adaptation, and post-stress recovery [190]. ABA also contributes to redox homeostasis by modulating ROS, influencing stomatal closure and root architecture [191,192]. Other hormones, including auxin, brassinosteroids, and gibberellins, might further shape ROS–NO signaling, adding additional layers of hormonal regulation [191].

The integration of these multiple signals is implicated in specific morphological responses to waterlogging. We will summarize them in the section below.

### 3.3. ROS and NO-Dependent Signals Involved in Formation of Aerenchyma, Adventitious Roots, and ROL Barrier

Under waterlogging, lysigenous aerenchyma—air-filled channels formed via programmed cell death (PCD)—develop in roots and stems to facilitate internal oxygen diffusion. PCD is well established as a ROS-dependent process, with mechanisms highly conserved across species. In wheat and other cereals, ethylene and ROS act synergistically: ethylene stimulates NADPH oxidases (RBOHs), generating ROS that trigger cortical cell death and aerenchyma formation [193]. In sunflowers, waterlogging- and ethylene-induced ROS-mediated PCD is essential for aerenchyma development, and inhibition of ROS production markedly reduces its formation [194]. Ca^2+^-dependent signaling is also integrated with ROS in rice. Li and co-workers demonstrated that CDPK5 and CDPK13 activate ROS production via phosphorylation of serine residues in RBOHH [195]. Knockout of both CDPKs nearly abolishes adventitious root formation, highlighting the significance of this Ca^2+^ pathway. NO further contributes to PCD regulation. In wheat roots under hypoxia, NO produced via nitrate reductase enhances ethylene biosynthesis and ROS accumulation, promoting PCD and aerenchyma development, whereas NO scavenging suppresses these processes [56]. In rice, ONOO^−^ formed from NO and superoxide, is critical for ethylene-mediated aerenchyma initiation, as scavenging ONOO^−^ blocks formation even in the presence of ethylene donors [196]. As noted above, ROS and NO signaling are tightly linked with ERF-VII transcription factors. In maize, EREB180 facilitates both aerenchyma formation and the activity of ROS-scavenging enzymes [170].

Waterlogging frequently induces adventitious root (AR) growth, often accompanied by aerenchyma to facilitate supplemental oxygen transport. In the halophyte Suaeda salsa, SNPs promote AR initiation and maintain cell viability, whereas NO scavengers inhibit these effects, highlighting NO’s direct signaling role [55]. In wheat, waterlogging “priming” upregulates transcripts encoding RBOH and ethylene biosynthesis enzymes, 1-AMINOCYCLOPROPANE-1-CARBOXYLATE SYNTHASE 2 and 4 (ACS2, ACS4), enhancing ROS production, AR proliferation, aerenchyma formation, and activities of anaerobic respiratory enzymes, such as ADH and PDC, thereby increasing ATP synthesis [197].

Recent studies suggest that PCD-related mechanisms are also integrated with AR formation [198]. In poplar, the transcription factor MYB180 was identified as a key regulator of AR development; both dominant repression and overexpression of MYB180 significantly reduced AR numbers. MYB180 regulates PCD in root cortex cells, associated with elevated ROS levels and altered expression of genes involved in ROS metabolism, PCD, and ethylene synthesis [198]. In cucumber, transgenic plants overexpressing Prx73, which encodes PEROXIDASE73, exhibit enhanced AR formation under waterlogging, whereas silencing Prx73 impairs AR development [199]. In this context, ERF7-3, a waterlogging-responsive ERF transcription factor, directly binds the ATCTA-box motif in the *Prx73* promoter to activate its expression. Notably, AR enhancement in transgenic plants coincides with increased ROS-scavenging activity, emphasizing the importance of fine-tuning ROS levels in coordinating AR formation and PCD under waterlogging.

To minimize oxygen loss to anoxic soil, many species develop a suberin-rich exodermis, the radial oxygen loss (ROL) barrier, in adventitious roots. In rice, ABA mediates this process, inducing exodermal suberization, while ABA inhibition or mutation impairs ROL formation [200]. Although direct roles of ROS or NO in ROL barrier formation are less documented, the coordinated development of adventitious roots, aerenchyma, and the ROL barrier suggests integration of ROS/NO signaling with hormone-driven suberization.

Overall, the formation of aerenchyma, adventitious roots, and the ROL barrier represents a coordinated anatomical adaptation to waterlogging. Ethylene-induced ROS trigger PCD and aerenchyma, with NO enhancing ethylene and ROS accumulation. Adventitious root emergence is promoted by NO, ROS, and ethylene, while ABA strengthens root surfaces through suberin deposition. The interplay of redox signaling and hormone regulation orchestrates root anatomical remodeling, optimizing oxygen transport and improving plant survival under waterlogged conditions.

## 4. Signaling Pathways Involved in ROS- and NO-Mediated Plant Responses to Combined Salinity and Waterlogging Stresses

### 4.1. Sources of ROS and NO Under Combined Salt and Waterlogging Stress

Under combined salinity and waterlogging, ROS generation is spatially distributed across multiple organelles and compartments, and the relative contributions may shift compared to single stresses. Under saline conditions, especially in illuminated leaves, electron transport in PSII/PSI can leak electrons to O_2_ via the Mehler reaction, forming superoxide and subsequently H_2_O_2_. Impaired CO_2_ assimilation under salt may exacerbate over-reduction and ROS formation in chloroplasts [17]. During hypoxia, mitochondrial electron transport becomes constrained. Under low O_2_, the electron transport chain (especially complexes I and III) may leak electrons to residual O_2_ or alternate acceptors, producing ROS. Upon reoxygenation, a burst of mitochondria-derived ROS is common [201]. In combined stress, mitochondrial ROS may be further aggravated as energy metabolism is pushed to its limits. Oxidases (e.g., glycolate oxidase, acyl-CoA oxidase) in peroxisomes also generate H_2_O_2_ during oxidative metabolism (e.g., photorespiration or fatty acid β-oxidation). Under stress, flux through peroxisomal pathways may increase, contributing to ROS load. Some ROS may be generated in the cytosol by yet less well-defined enzymes (e.g., oxidases and oxidant-producing metabolic reactions). Also, transition-metal catalyzed Fenton chemistry (Fe^2+^ + H_2_O_2_ →·OH) can generate hydroxyl radicals in diverse compartments if H_2_O_2_ and Fe^2+^ coexist.

One of the earliest ROS sources is via plasma membrane-localized RBOHs, which use NADPH to reduce O_2_ to O_2_^−^. This apoplastic ROS may function as an extracellular signal, initiating downstream cascades of Ca^2+^ influx, cell wall modification, and systemic signaling [202]. In the context of combined stress, the apoplastic RBOH-driven ROS may act as the “first wave,” while chloroplast and mitochondrial ROS provide internal redox cues and exacerbate oxidative pressure. One challenge under combined salt and waterlogging is that hypoxia reduces electron acceptor availability in mitochondria, thereby increasing electron leak and ROS formation, while salt exacerbates electron pressure in chloroplasts and plasma membrane compartments. The sum of these sources may push the antioxidant buffering capacity toward saturation.

Nitric oxide in plants arises from multiple enzymatic and non-enzymatic pathways, and under combined salt and waterlogging stress, the balance among these sources can shift. In many species, NR can reduce nitrite (NO_2_^−^) to NO (especially under low nitrate or under certain regulatory states). Under salt stress, NR-dependent NO production is well documented [203]. During waterlogging/hypoxia, NR may operate under altered redox and substrate conditions to modulate NO flux. Under hypoxic conditions, nitrite can act as an alternative electron acceptor in mitochondria. In low O_2_, electrons from the mitochondrial electron transport chain may reduce nitrite to NO (i.e., site III/IV engaged in nitrite -> NO) [204]—in effect, a partial hypoxic respiration bypass. This route becomes more prominent under flooding stress. Although canonical NOS has not been conclusively established in plants, there is evidence for NOS-like activity under certain stress contexts, possibly offering a regulated NO source [205]. In addition, it was also demonstrated that, under hypoxia, NO can induce S-nitrosylation of GSNOR, leading to its recruitment into the autophagosome and degradation [206]. Such regulatory mechanisms of GSNOR might also contribute to fine modulation of NO level under waterlogging or related stresses. Regulation of NO under combined stresses must adjust to varying oxygen, substrate (nitrite/nitrate), redox, and enzyme activity constraints. Because NO is short-lived (half-life of seconds), its spatial and temporal production must be tightly regulated. NO homeostasis is managed not only by generation but also through scavenging (e.g., non-symbiotic hemoglobins, S-nitrosoglutathione reductase, peroxiredoxins, etc.) [207]. In combined salt and waterlogging stress, it can be proposed that under hypoxic root zones, mitochondrial nitrite reduction may become an important NO source, whereas in shoots (non-hypoxic), NR and apoplastic routes may prevail. The interplay between these sources and oxidative stress is crucial for NO, functioning as a fine-tuner of stress responses.

### 4.2. ROS- and NO-Related Signals Underlying Responses of Plants to Salt Stress and Waterlogging Applied Individually or in Combination

**Figure 4:**
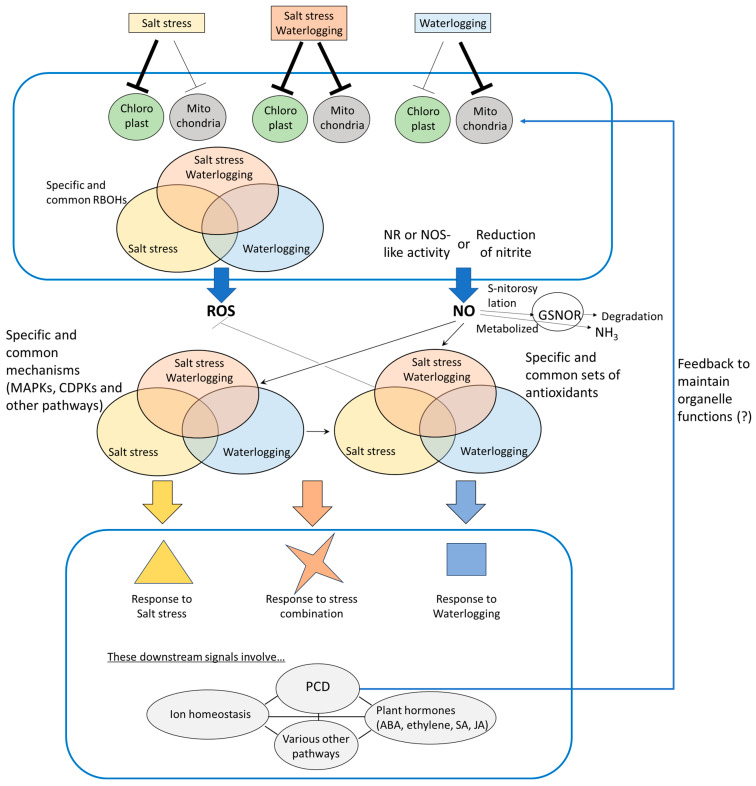
Overview of signals regulating responses of plants to salt stress, waterlogging, and their combination. Effects of stress on organelles can be combined under the stress combination, and several common mechanisms may function to counteract these effects. However, mechanisms that specifically operate under single or combined stresses may exist. These common and specific mechanisms might be established via fine-tuning of coordination among multiple stresses regulated by ROS and NO.

When plants are exposed to salinity and waterlogging stress simultaneously, they often experience exacerbated oxidative stress due to both osmotic and/or ionic imbalance. This exacerbated stress leads to elevated production of different ROS—including superoxide (O_2_^−^) and hydrogen peroxide (H_2_O_2_)—which surpass levels found under individual stress conditions [59]. In maize, for instance, combined salt and waterlogging stress was observed to trigger upregulation of enzymatic antioxidants such as SOD, CAT, and APX, highlighting a critical ROS-scavenging response [59]. These enzymatic defenses may help counterbalance oxidative damage while allowing for signaling functions of ROS under a combination of salt stress and waterlogging, as well as when each of them individually occurs (Table 1). However, it should be noted that the detailed mode of coordination among these ROS-scavenging systems under these single and combined stresses still needs to be elucidated. Interestingly, in tomato, oxidative damage caused by waterlogging was alleviated when salt stress was simultaneously applied [58]. The activities of various antioxidant enzymes were differentially modulated under salt stress, waterlogging, and their combination [58], suggesting that ROS-regulatory systems operate in a stress-specific and organelle-dependent manner (Table 1). In addition, the behavior of ROS-scavenging systems under individual and combined stresses varies among plant species [58,208,209,210]. It is likely that distinct genes encoding different isoforms of ROS-scavenging enzymes play significant roles under these conditions. Indeed, it has long been established that isoforms of antioxidant enzymes function in distinct subcellular compartments [211]. Given that salt stress primarily affects the photosynthetic apparatus, while respiratory processes are highly sensitive to waterlogging, specific ROS-scavenging mechanisms may be differentially activated in chloroplasts, mitochondria and other organelles depending on the types of stress (i.e., depending on individual or combined stresses, Figure 4). In addition, it is likely that CDPKs and MAPKs also play key roles in regulating specific responses to single or combined stresses. Although kinase-dependent signaling pathways are commonly involved in both salt stress and waterlogging, distinct sets of these kinases may function to fine-tune stress-specific mechanisms. Therefore, it would be intriguing to investigate which specific or shared CDPKs and MAPKs are activated under individual and combined stress conditions.

Nitric oxide (NO) plays a pivotal role in modulating plant responses to individual salinity stress, often intertwined with ROS and other signaling networks involving Ca^2+^, kinases, and plant hormones [15,212]. To some extent, these signaling networks may overlap under salt stress, waterlogging, and their combination (Figure 1 and Figure 3). One of the best-characterized outcomes of ROS–NO interplay involves post-translational modifications (PTMs) such as S-nitrosylation (the attachment of a NO moiety to cysteine thiols) and nitration (addition of a nitro group to tyrosine residues) [213]. These modifications regulate protein activity, localization, stability, and interactions. Key antioxidant enzymes, including APX, CAT, SOD, and enzymes in the ascorbate–glutathione cycle, undergo S-nitrosylation or nitration, which can alter their catalytic performance. For example, S-nitrosylation of APX modulates its activity in salt-stressed pea leaves, potentially enhancing enzyme stability or reactivity [214], whereas nitration typically inhibits APX activity. Similarly, RBOHs can be S-nitrosylated or otherwise redox-modified, affecting their ROS-generating capacity [215]. Through such feedback mechanisms, NO can suppress excessive ROS production by directly modulating RBOH activity. Beyond antioxidant enzymes, numerous kinases, transcription factors, ion channels, and transporters are targets of S-nitrosylation. For instance, NO-mediated S-nitrosylation of ROP2 in Arabidopsis influences auxin transport and root growth. NO can also regulate its own metabolic enzymes through PTMs, such as S-nitrosylation of non-symbiotic hemoglobins or peroxiredoxins, thereby modulating NO scavenging efficiency. Under combined salinity and waterlogging, the coexistence of elevated ROS and fluctuating NO levels may shift the balance of PTMs. Excess ROS can induce oxidative modifications (e.g., sulfenylation or disulfide formation) that compete with or disrupt nitrosylation events. Moreover, compartmental specificity is critical: enzymes in hypoxic roots may undergo distinct redox modifications compared with their counterparts in oxygenated tissues. Altogether, ROS and NO engage in a highly coordinated interplay—NO mitigates ROS overaccumulation while generating reactive nitrogen species (RNS), and ROS in turn regulate NO production and turnover. These reciprocal PTMs act as molecular switches linking ROS–NO crosstalk to adaptive physiological responses.

**Table 1:** Oxidative, antioxidative, and other responses of different crop species to salt stress, waterlogging stress, or their combination. Up and down arrows indicate up- and down-regulation of mechanisms, respectively.

Plant Species	Stress Type & Duration	Oxidative Responses	Antioxidative/Other Responses	Reference
*Cicer arietinum* L.	NaCl 100 mM; 45 d	MDA, H_2_O_2_ ↑	SOD, CAT, APX, GR ↑	[6]
*Lycopersicum esculentum* L. cv. Micro-tom	NaCl 50 mM + waterlogging; 14 d	MDA, H_2_O_2_ ↑	CAT, GPX, GST ↓ APX, MDHAR, DHAR, GR ↓	[58]
*Zea mays*	NaCl 10 dSm^−1^ + waterlogging; 7 d	H_2_O_2_ ↑	SOD, CAT, APX ↑	[59]
*Suaeda glauca*	NaCl 400 mM; 10 d	MDA, O_2_^•−^, H_2_O_2_ ↑	SOD, APX ↓	[60]
*Elaeagnus angustifolia*	NaCl 0.6% + waterlogging; 14 d	MDA, H_2_O_2_ ↑	SOD, APX, POD, GR ↑	[62]
*Cucumis sativus*	Waterlogging; 5 d	MDA, H_2_O_2_, NO ↑	NR activity ↑ RBOH9, NRT1.8, REP2.3, HEM3 ↑	[183]
*Arabidopsis thaliana* L.	Anoxia; 4 h	H_2_O_2_, NO ↑	ASC ↓ DHA↑ GR, POD, CAT, APX ↓	[202]
*Momordica charantia*	NaCl 25 mM; 7 d	MDA, O_2_^•−^, H_2_O_2_, NO ↑	CAT, APX, GR, GST ↑	[209]
*Cajanus cajan* L. Millsp.	NaCl 30 mM + waterlogging; 12 d	Membrane injury ↑ Lipid peroxidation ↑	Proline ↑	[214]
*Mentha aquatica* L.	NaCl 150 mM + waterlogging; 30 d	MDA, H_2_O_2_ ↑	SOD, CAT, APX ↑	[215]
*Triticum aestivum*	NaCl 195 mM + waterlogging; 5 d	MDA, O_2_^•−^, H_2_O_2_ ↑	SOD, CAT, APX ↓	[216]

Both enzymatic and non-enzymatic ROS-scavenging systems are commonly activated via NO signaling under salt stress, waterlogging, and their combination. Therefore, ROS–NO crosstalk can serve as an effective anchor mechanism for modulating responses of plants to these stresses. Signal specificity is likely influenced by stress-dependent regulation of ROS and NO production. As noted above, the primary mechanisms controlling ROS and NO levels may operate in different organelles depending on the type of stress (see Section 4.1, [17,201,203,204,205,206]). Therefore, it is essential to consider the stress-specific coordination of ROS and NO production within cells under both individual and combined stress conditions.

The coordination of ROS–NO crosstalk may also shape downstream signaling pathways under salt stress, waterlogging, and their combination, thereby protecting plant organs, tissues, and cells from stress-induced damage. As described above, salt stress activates Na^+^ extrusion via SOS-dependent ion homeostasis and osmotic stress responses through ABA-mediated stomatal closure and osmolyte accumulation (see Section 2.3), whereas waterlogging induces morphological adaptations such as PCD-dependent aerenchyma formation and adventitious root development via ethylene signaling (see Section 3.3). Under combined salt stress and waterlogging, both ABA- and ethylene-mediated responses are likely activated, as these hormones are key regulators of plant responses to abiotic stresses [31,49], and their associated signaling components may be further modified when both stresses occur simultaneously. It is essential to uncover specific mechanisms integrated with these hormone signals under a combination of salt stress and waterlogging.

In addition, other plant hormones such as SA and JA may also play important roles in responses to salt stress and waterlogging that occur individually and in combination. Although the functions of these plant hormones in tolerance to waterlogging and their combination with salt stress remain unclear, it is plausible that they contribute to PCD regulation under such conditions. SA and JA are well known as regulators of PCD during defense responses and may act as downstream components of ROS–NO-mediated signaling [216]. Moreover, both hormones are implicated in modulating chloroplast retrograde signaling to regulate photosynthetic activity [217]. Therefore, future studies should investigate how hormone-mediated PCD regulation and feedback mechanisms that maintain organelle function are integrated under salt stress, waterlogging, and their combined occurrence.

Overall, ROS and NO are engaged in a tightly coordinated interplay: NO mitigates ROS overaccumulation while generating reactive nitrogen species, and ROS, in turn, influence NO production and turnover. These reciprocal PTMs act as molecular switches linking ROS–NO crosstalk to adaptive physiological responses. Given the central role of ROS regulation in plant adaptation to salt and waterlogging stresses, it is likely that ROS–NO interactions are finely tuned depending on the specific stress or their combination. As discussed above, salt stress primarily affects photosynthetic activity, whereas waterlogging strongly impacts mitochondrial metabolism. Thus, ROS–NO crosstalk may be crucial for regulating energy metabolism under these conditions by modulating kinase activities and the production of energy-related metabolites such as sugars, amino acids, and osmolytes. Elucidating how these signaling pathways integrate to coordinate organelle function and energy metabolism represents an important frontier in understanding plant resilience to combined environmental stresses.

## 5. Conclusions

Excessive ROS generated under salt stress, waterlogging, and their combination is one of the primary causes of damage to a wide range of vital plant processes. Therefore, efficient ROS scavenging is crucial for protecting plants against these individual and combined stresses. At the same time, ROS and NO signals—and their integration with other signaling pathways involving Ca^2+^, kinases, and transcription factors—are essential for regulating plant responses to these stresses. Thus, maintaining appropriate ROS levels through a balance of production and scavenging is fundamental for stress adaptation. Although this concept applies to both individual and combined stresses, distinct mechanisms are likely to operate under each condition. One of the most important questions to address is how signals originating from different organelles are coordinated. As mentioned above, while both salt stress and waterlogging affect chloroplast and mitochondrial functions, the nature and extent of these effects differ between the two stresses. Therefore, elucidating how ROS-regulatory systems and associated signaling pathways are integrated across organelles will be key to answering this question. It is also of great interest to explore how chloroplast and mitochondrial retrograde signaling pathways are coordinated under salt stress, waterlogging, and their combination. Although the roles of these signals remain poorly understood, their significance is evident from the physiological effects of these stresses. Indeed, previous studies have proposed functional integration between chloroplast and mitochondrial signaling under abiotic stress [218]. Moreover, plant protection against salt stress, waterlogging, and their combination depends on maintaining homeostasis at both cellular and whole-plant levels. Consequently, investigating stress-specific mechanisms across different organs, tissues, and organelles—including those beyond chloroplasts and mitochondria—will be essential for advancing our understanding of plant responses to individual and combined environmental stresses.

## Data Availability

No new data were created or analyzed in this study. Data sharing is not applicable to this article.

## Abbreviations

The following abbreviations are used in this manuscript:

AA	ascorbic acid
ABA	abscisic acid
ADH	alcohol dehydrogenase
AOX	ascorbate oxidase
APX	ascorbate peroxidase
ASA	ascorbate
BADH	betaine aldehyde dehydrogenase
BAM	barely any meristem
CAT	catalase
CDLC	calcium-dependent protein kinases
CHC	clathrin heavy chain
CLC	clathrin light chain
DHAR	dehydroascorbate reductase
ERF	ethylene-responsive factor
FLS	flagellin sensitive
GABA	γ-aminobutyric acid
GB	gibberellin
GBF	g-box binding factor
GR	glutathione reductase
GSH	glutathione
GSNOR	S-nitrosoglutathione reductase
GST	glutathione s-transferase
HB	hemoglobin
MDA	malondialdehyde
MDHAR	monodehydroascorbate reductase
MJ	methyl jasmonate
NADPH	nicotinamide adenine dinucleotide phosphate
NF	nuclear factory
NO	nitric oxide
NR	nitrate reductase
OST	open stomata
PDC	pyruvate decarboxylase
PIF	phytochrome interacting factor
POD	peroxidase
RBOH	respiratory burst oxidase homolog
RNS	reactive nitrogen species
ROS	reactive oxygen species
SOD	superoxide dismutase
SOS	salt overlay sensitive
TPC	two pore channel
